# Association of milk components with intra-mammary inflammation in Jaffrabadi buffaloes

**DOI:** 10.14202/vetworld.2015.989-993

**Published:** 2015-08-18

**Authors:** T. K. Patbandha, S. Marandi, K. Ravikala, R. Pathak, B. R. Maharana, K. S. Murthy

**Affiliations:** 1 Polytechnic in Animal Husbandry, College of Veterinary Science and A.H. Junagadh Agricultural University, Junagadh - 362 001, Gujarat, India; 2 Instructional Livestock Farm Complex, College of Veterinary Science and A.H. Junagadh Agricultural University, Junagadh - 362 001, Gujarat, India; 3 College of Agriculture and Research Station, Janjgir-Champa - 495 668, Chhattisgarh, India; 4 Department of Veterinary Parasitology, College of Veterinary Science and A.H. Junagadh Agricultural University, Junagadh - 362 001, Gujarat, India; 5 Cattle Breeding Farm, Junagadh Agricultural University, Junagadh - 362 001, Gujarat, India

**Keywords:** buffaloes, intra-mammary inflammation, milk components, milk lactose

## Abstract

**Aim:**

To study the alteration of major milk components such as milk fat, protein, lactose, solid not fat (SNF) and total solids (TS) and their association with different degree of intra-mammary inflammation (IMI) in Jaffrabadi buffaloes.

**Materials and Methods:**

Milk samples (n=1516) were collected from Jaffrabadi buffaloes separately from each quarter. Milk samples were analyzed for milk fat, protein, lactose, SNF and TS percent on the same day using milk analyzer “LACTOSCAN.” Milk samples were checked for IMI by California mastitis test (CMT), and the results were expressed as negative (0), +, ++, and +++ CMT score. The traits of milk components which showed significant difference (p<0.05) between samples from inflamed and non-inflamed quarters were analyzed by receiver operating characteristic (ROC) analysis to see the accuracy and degree of association with IMI.

**Results:**

Among several milk components, milk protein and lactose percent showed a significant difference (p<0.05) between milk samples from normal and inflamed quarters. Though, during the early stage of mammary gland inflammation milk protein percent remained significantly high (p<0.05), later with an increase in the degree of severity of inflammation it did not show any difference. Milk samples from normal udder quarters had significantly higher lactose percent than inflamed quarters (p<0.05). Milk lactose percent decreased gradually with an increase in the degree of severity of inflammation. ROC analysis revealed that milk samples having lactose content below the threshold values had significantly higher chances to come from inflamed udder quarters (p<0.05). Though, the value of the area under curve (AUC) indicated that milk lactose was significantly associated with IMI (p<0.05), the accuracy was moderate (AUC=0.71-0.75).

**Conclusions:**

The results of the present study indicated that milk lactose percent gradually and significantly reduced during IMI and can be used as a marker for identification of IMI in buffaloes. However, ROC analysis further confirmed that using milk lactose IMI can be identified with moderate accuracy.

## Introduction

Mastitis in buffaloes is an important production disease which causes huge economic losses to dairy industry in terms of production loss, milk loss due to disposal after treatment, treatment loss, man power loss as well as premature culling. The losses owing to mastitis in India was first reported Rs. 52.9 crore annually in the year 1962, which reached to Rs. 6053.21 crore annually in the year 2001 [1,2]. It has been observed that treatment cost of mastitis accounts Rs. 73.00/day and complete fibrosis of a single quarter on an average decrease market value of buffalo by Rs. 2500 [3]. In India, the incidence of subclinical mastitis was higher (10-50% in cows and 5-20% in buffaloes) compared to clinical mastitis (1-10%) [4].

Milk composition is considered as an important factor for the dairy farmers to maintain raw milk quality, dairy industries to produce better quality dairy products and consumers to maintain nutritional quality and safety [5]. There are several factors which are broadly categorized into environmental, nutritional, physiological, and pathological which significantly contribute variation of milk components (fat, protein, lactose, solid not fat [SNF] and total solids [TS]). Among these, fat is being considered as the most variable component, followed by protein, lactose is being the least [6,7]. Though, better managerial practices could reduce the effect of environmental and nutritional stress on milk composition, the effect due to physiological condition (such as stage of lactation) could not be altered. Further, during intra-mammary inflammation (IMI), alteration of milk lactose occurs markedly, other traits such as protein, SNF and TS show low to moderate variation and milk fat least variable [5,8-10]. Therefore, it is important to study the alteration of milk components during IMI.

Though, alteration of milk components during IMI has been studied well in dairy cattle, the information on buffalo particularly Jaffrabadi buffaloes is scanty. Further, information regarding accuracy and degree of association of milk components with IMI are still lacking. With this backdrop, the present study was designed to see the alteration of milk components with IMI and further to study the accuracy and degree of association of milk components with IMI in Jaffrabadi buffaloes.

## Materials and Methods

### Ethical approval

The experimental procedures carried out in the present study were approved by the Institutional Animal Ethics Committee, College of Veterinary Science and A.H., Junagadh Agricultural University.

### Experimental animals and management

The experiment was carried out on milk samples of Jaffrabadi buffaloes (2^nd^-4^th^ lactation with previous 305 days milk yield about 1800-2000 kg) at Junagadh Agricultural University, Junagadh (Gujarat), India between March 2014 and February 2015. Junagadh is located in South Saurashtra agroclimatic region of Gujarat and receives 625-750 mm of average annual rainfall, and the climate is dry to sub-humid. The experimental buffaloes were maintained in loose housing system of management on pucca flooring at Cattle Breeding Farm of the University. The buffaloes were fed *ad libitum* amount of green and dry fodder according to availability of the season. The buffaloes were fed mixture of cottonseed cake (crude protein [CP]: 35% and total digestible nutrients [TDN]: 75%), ground maize (CP: 10% and TDN: 85%) and Amul dan (CP: 22% and TDN: 70%) during milking. Milking was done at the milking parlor at 4.00 am and 4.00 pm and for milk let down, suckling of the calf was practiced. Splashing was practiced to reduce heat stress of the milking buffaloes during the summer season at 10-11 am and again before afternoon milking. All managerial practices were followed uniformly throughout the year to reduce the environmental effect on the animals.

### Sampling procedure and analysis of milk components

A total of 1516 quarterly milk samples were taken at afternoon milking from Jaffrabadi buffaloes with 480, 404 and 631 samples during early (≤100 days in milk), mid (101-200 days in milk) and late (>200 days in milk) lactation, respectively. Milk samples were analyzed on the same day for milk fat, protein, lactose, SNF and TS percent by milk analyzer “LACTOSCAN” (New Dairy Engineering and Trading Company Pvt. Ltd., Delhi, India).

### California mastitis test

The California mastitis test (CMT) test is a gold standard cow side test found to be suitable for herd health monitoring programs to detect inflammation of mammary glands [11]. Hence, for IMI samples were checked by CMT test and the results were expressed as negative (0), +, ++, and +++ CMT score.

### Statistical analysis

The data sets were analyzed by one-way analysis of variance to see the effect of different degree of IMI on various traits of milk components (milk fat, protein, lactose, ash, SNF, and TS percent). Tukey *post-hoc* test was used to compare all pairwise differences in mean and difference was considered as significant if p<0.05. As milk lactose from inflamed and non-inflamed quarters showed particular trend, as well as significant difference (p<0.05), hence further analyzed by receiver operating characteristic (ROC) analysis using Sigmaplot 11 software package (Systat Software, Inc., California, USA) to see the accuracy and degree of association with IMI. The ROC analysis produces an area under the curve (AUC), threshold value and their corresponding sensitivity (Se), specificity (Sp) and positive likelihood ratio (LR+) value [12,13]. The AUC is a two-dimensional graph (Se plotted in Y-axis and 1-Sp plotted in X-axis) which interprets the accuracy of the diagnostic test and used to distinguish the inflamed and non-inflamed udder quarters. Though, different threshold values were produced by ROC analysis; the value having highest corresponding combined Se and Sp was called as a critical threshold value for identification of IMI. The Se was defined as the proportion of milk samples from inflamed quarters having milk lactose below the threshold value (exposed) and the Sp was the proportion of normal milk samples having milk lactose above the threshold value (unexposed). The LR+ was defined as a number of times samples having milk lactose below the threshold value (exposed) were more likely to come from inflamed quarters.

## Results and Discussion

In the present study, 10.0, 10.9 and 8.4% quarterly milk samples were observed to be positive for different degree of IMI during early, mid and late lactation (Table-1) with overall incidence 9.56% (145/1516). The overall incidence of IMI (9.7%) in Jaffrabadi buffaloes is almost similar to Joshi and Gokhale [4], who reported incidence of subclinical mastitis 5-20% and clinical mastitis 1-10% in buffaloes in India. However, Sudhan and Sharma [1] and Sharma *et al*. [2] reported that prevalence of mastitis in buffaloes varies from 7.9% to 68.6% in India. The lower incidence of mastitis observed in the present study might be contributed by better housing and managerial practices adopted in the farm.

**Table-1:** Incidence of IMI of Jaffrabadi buffaloes.

Lactation stage	Total samples	CMT score (%)				Total positive samples (%)

		0	+	++	+++	
Early	480	432	12 (2.5)	18 (3.7)	18 (3.7)	48 (10.0)
Mid	404	360	12 (2.9)	14 (3.5)	18 (4.5)	44 (10.9)
Late	632	579	8 (1.3)	19 (3.0)	26 (4.1)	53 (8.4)

CMT=California mastitis test, IMI=Intra-mammary inflammation

The mean values of different milk components (milk fat, protein, lactose, SNF and TS) were depicted in Table-2. Mean values of different major milk components irrespective of IMI were more or less comparable with the previous reports in Jaffrabadi buffaloes [14-16] and other breeds of buffaloes [17-19]. We observed significant alteration of milk components particularly milk lactose followed by milk protein between inflamed and non-inflamed udder quarter (p<0.05). Milk lactose percent was significantly higher in normal milk samples compared to the samples from inflamed quarters and decreased significantly (p<0.05) with the advancement of the severity of inflammation (Table-2). Previous studies consistently reported that milk of inflamed quarter had significantly lower lactose content compared to non-inflamed quarter [5,8-10,17], and same being observed in our study. Lower milk lactose content during IMI may be attributed to impairment of the synthetic activity of mammary tissues or decomposition by leucocytes or mastitis causing microbes or leakage from milk into blood [5,10,20]. Hence, lactose could be used as indicator due to consistent change during IMI [5]. Milk protein percent remained significantly high (p<0.05) in mild inflammatory condition compared to samples from normal quarters and later decreased significantly with an increase in the severity of inflammation (Table-2, p<0.05). Bansal *et al*. [8] reported higher milk protein content in milk samples from inflamed quarters of cows than healthy quarters and supported by our study. However, in Murrah buffaloes, Bansal *et al*. [17] observed lower milk protein percent in inflamed quarters compared to healthy quarters. In the present study, as the degree of severity of inflammation progress milk protein percent reduced and remained similar level as that of the healthy quarter. The mastitis causative pathogens and degree of inflammation also play a significant role in alteration of milk protein [5]. Further, mastitis decreases milk casein content whereas increases whey proteins, serum albumin and immunoglobulins. Thus, contributes to either increase or decrease or the same level of total proteins percent in milk during IMI [5,8-10].

**Table-2:** Least square mean±SEM of milk components and CMT score.

Lactation stage	CMT score			

	0	+	++	+++
Milk fat (g%)				
Early	7.66±0.13^A^	7.85±0.20^A^	7.82±0.38^A^	7.96±0.20^A^
Mid	8.19±0.14^A^	8.09±0.39^A^	8.63±0.33^A^	8.04±0.23^A^
Late	9.00±0.13^A^	9.52±0.14^A^	8.61±0.31^A^	9.43±0.28^A^
Milk protein (g%)				
Early	4.20±0.05^A^	4.75±0.13^B^	4.25±0.11^AB^	4.20±0.15^A^
Mid	4.17±0.06^A^	3.96±0.05^A^	4.05±0.15^A^	4.15±0.09^A^
Late	4.50±0.06^A^	4.80±0.13^A^	4.35±0.11^A^	4.37±0.11^A^
Milk lactose (g%)				
Early	5.65±0.08^A^	5.41±0.23^AB^	5.22±0.12^AB^	5.13±0.12^B^
Mid	5.38±0.08^A^	5.14±0.07^AB^	5.12±0.22^AB^	4.89±0.07^B^
Late	5.93±0.08^A^	6.01±0.38^AB^	5.29±0.18^B^	5.21±0.07^B^
Milk SNF (g%)				
Early	10.82±0.13^A^	11.15±0.30^A^	10.41±0.23^A^	10.25±0.27^A^
Mid	10.48±0.14^A^	9.98±0.13^A^	10.06±0.36^A^	9.96±0.11^A^
Late	11.42±0.14^A^	11.77±0.41^A^	10.58±0.26^A^	10.55±0.15^A^
Milk TS (g%)				
Early	18.47±0.23^A^	19.00±0.37^A^	18.23±0.55^A^	18.21±0.36^A^
Mid	18.67±0.24^A^	18.80±0.49^A^	18.69±0.64^A^	18.00±0.22^A^
Late	20.42±0.25^A^	21.28±0.39^A^	19.19±0.54^A^	19.98±0.37^A^

Means with different superscript (A, B) within a row differ significantly (p<0.05), SEM=Standard error of mean, CMT=California mastitis test, SNF=Solid not fat, TS=Total solids

Milk fat percent did not differ between milk samples from inflamed and non-inflamed quarters (Table-2) which is in agreement with Malek dos Reis *et al*. [5]. However, other studies reported lower content of milk fat during udder inflammation [8,10]. The variation of results may be associated with the degree of inflammation or causative agents [5]. Malek dos Reis *et al*. [5] observed that milk SNF and TS reduced significantly during IMI in dairy cows but such difference was not observed in our study. Further, lower milk SNF percent was observed by Bansal *et al*. [8] and Hussain *et al*. [20] in inflamed quarters than healthy quarters of buffaloes. The milk SNF and TS were not affected by single milk traits, rather except fat, the milk protein, lactose, ash, etc. together affect milk SNF and then with fat affect milk TS. Hence, it is difficult to interprete the alteration of milk SNF and TS during inflammation of mammary glands.

Lactose content of milk consistently and significantly reduced with the advancement of the severity of IMI in Jaffrabadi buffaloes. Hence, milk lactose was further analyzed by ROC analysis to distinguish healthy and inflamed udder quarters of buffaloes accurately and to associate with the different degree of IMI. The AUC, threshold value, Se, Sp and LR+ of milk lactose during different lactation stage with a different degree of inflammation are presented in Table-3. The AUC of ROC analysis revealed that milk lactose could be used to distinguish inflamed and healthy quarters with 71-75% accuracy (AUC=0.71-0.75). The AUC is considered as a good measure of accuracy to distinguish inflamed and non-inflamed udder quarters as it does not depend on the threshold value or the prevalence of disease or inflammation [21]. Bansal *et al*. [8] reported that milk lactose content in cows can be used to classify 81% of inflamed and non-inflamed udder quarters correctly. They also reported that milk lactose was more accurate to discriminate inflamed and non-inflamed quarters compared to milk electrical conductivity (69% accuracy) and pH (59% accuracy). Similarly, using milk lactose in buffaloes, Bansal *et al*. [17] observed 83.76% accuracy for differentiation of healthy and diseased quarters, which was higher than the other milk traits such as SNF (55.08% accuracy) and electrical conductivity (62.94% accuracy). The value of AUC as 0.71-0.75 indicated that milk lactose is moderately accurate to distinguish inflamed and healthy udder quarters in Jaffrabadi buffaloes [13]. The threshold values of milk lactose varied from 4.97 to 5.48 g% during different stage of lactation depending on severity of inflammation which are comparatively higher than Bansal *et al*. [8] i.e. 4.8 g% may be attributed by species and breed of dairy animals. However, the threshold values of milk lactose observed in the present study are comparatively lower than Bansal *et al*. [17], who reported 5.5 g% as threshold value for identification of mastitis in Murrah buffaloes. LR+ indicated the number of times a sample would come from inflamed quarters if milk lactose content remained below the threshold values. In the present study, there was 2.19-2.53 times higher chance that a milk sample having lower milk lactose (below the threshold vale) was from inflamed quarters. The milk samples from late lactation having milk lactose <5.28 g% (exposed group) had comparatively higher chance to come from moderately inflamed udder quarter (LR+ = 2.53) than other periods. LR+ indicated that lower milk lactose is more associated with inflamed quarters.

**Table-3:** Threshold value and accuracy of milk lactose (g%) for IMI prediction.

Lactation stage	AUC	p-value	Threshold value	Se (%)	Sp (%)	LR+
Early lactation						
Normal versus severe	0.72	0.001	5.18	72.22	71.3	2.51
Mid lactation						
Normal versus severe	0.73	<0.001	4.97	66.67	72.22	2.39
Late lactation						
Normal versus moderate	0.71	0.002	5.28	68.42	73.06	2.53
Normal versus severe	0.75	<0.001	5.48	92.31	57.86	2.19

AUC=Area under curve, Se=Sensitivity, Sp=Specificity, LR+=Positive likelihood ratio, IMI=Intra-mammary inflammation

## Conclusion

Taken together, from the above results we concluded that among several milk components, only milk lactose followed a particular trend during IMI. Hence, milk lactose could be used as a marker for IMI in Jaffrabadi buffaloes. Milk samples with milk lactose below the threshold value had a higher chance to come from inflamed udder quarter. However, AUC of ROC analysis revealed that milk lactose is moderately accurate to differentiate inflamed and non-inflamed udder quarters in Jaffrabadi buffaloes.

## Authors’ Contributions

TKP, KR, and KSM designed the study. TKP and SM conducted the study, collected samples and analyzed the data. TKP, RP, and BRM drafted and revised the manuscript. All authors read and approved the final manuscript.
